# Mapping of nutrition policies and programs in South Asia towards achieving the Global Nutrition targets

**DOI:** 10.1186/s13690-023-01186-0

**Published:** 2023-09-19

**Authors:** Nidhi Wali, Kingsley Emwinyore Agho, Andre M. N. Renzaho

**Affiliations:** 1 School of Social Sciences, Western Sydney University, Locked Bag 1797, Penrith, NSW 2751 Australia; 2 School of Health Sciences, Western Sydney University, Campbelltown Campus, Locked Bag 1797, Penrith, NSW 2571 Australia; 3 African Vision Research Institute, Westville Campus, University of KwaZulu-Natal, Durban, South Africa; 4 School of Medicine, Translational Health Research Institute, Western Sydney University, Locked Bag 1797, Penrith, NSW 2571 Australia

**Keywords:** Global nutrition targets, Nutrition-specific, Nutrition- sensitive, Programs, Policies, Undernutrition, Overnutrition, South Asia, Sustainable development goals

## Abstract

**Background:**

South Asia continues to host the triple burden of child malnutrition with high levels of child undernutrition, hidden hunger (micronutrient deficiencies) and childhood overweight/obesity. To combat malnutrition, the international community along with the National governments have launched initiatives to track the country’s progress towards achieving the Global Nutrition targets by 2025. This review captures the country-specific efforts of nutrition-specific and nutrition-sensitive sectoral programs and policies towards achieving these targets for eight South Asian countries.

**Methods:**

A systematic internet search was undertaken to search relevant policies and programs from Government websites and twelve International Organisations working in the region. The authors developed a template to map the policies against the following criteria: (i) enabling supportive environment; (ii) Initiatives targeted at nutrition-specific interventions; and (iii) Initiatives targeted at nutrition-specific interventions that impact child malnutrition. A narrative descriptive approach was used to present findings.

**Results:**

All eight countries had relevant policies and programs to address child malnutrition and macronutrient deficiencies with targets for significant reductions in stunting and improved breastfeeding. However, despite the outlined there are major challenges of implementation, monitoring, evaluation and quality that persist with increased dependency on international donors and organisations for funding and/or implementation of nutrition plans.

**Conclusion:**

There is a need to contextualise efforts designated to donors and governments to improve the tracking of efforts that impact nutrition.

**Supplementary Information:**

The online version contains supplementary material available at 10.1186/s13690-023-01186-0.

**Table:** 

**Text box 1. Contributions to the literature**
• There is limited evidence of the policy coherence of South Asian countries with globally recommended actions to combat child malnutrition.
• This study generates evidence to inform country-wise efforts towards the Global Nutrition Targets.
• It mapped national nutrition-sensitive and specific policies and programs to address child malnutrition across the eight South Asia countries. Policies were mapped against a developed template, aligned with latest globally recommended actions by the World Health Organisation (WHO). This template can be a useful tool to track a country’s efforts in nutrition policy and practice.
• Findings highlight the challenges of national policy implementation and outreach.

## Introduction

South Asia has witnessed improvements in child undernutrition including stunting, wasting and underweight [1]. However, despite the observed improvements it continues to host triple burden of child malnutrition with high levels of child undernutrition, hidden hunger (micronutrient deficiencies) and childhood overweight/obesity [2, 3]. To encourage global, regional and country efforts to combat malnutrition, the international community has launched alliances, movements, initiatives and calls to action. In 2012, the World Health Assembly (WHA) of the World Health Organisation (WHO) launched the Comprehensive implementation plan (CIP) on maternal, infant and young child nutrition. This CIP specifies a set of six ambitious ‘Global Nutrition targets’ to achieve by 2025, these targets serve as a universal tool for tracking progress in nutrition. These Global Nutrition targets were considered in developing the Sustainable Development Goals (SDGs) Agenda 2030 [4] and are viewed as universal targets to track nutrition related progress. The SDGs endorsed in 2015 by the UN General Assembly sets nutrition as a key priority under goal no. 2, ‘End hunger, achieve food security and improved nutrition, and promote sustainable agriculture’, and goal 2.2, ‘ending all forms of malnutrition’ [5]. While nutrition is outlined in SDG2, nutrition is a critical component for achieving all other SDGs [6]. Initiatives such as the Scaling Up Nutrition (SUN) and actions for achieving the Global Nutrition targets and SDGs have been instrumental in mobilising committed actions across various stakeholders and the national government to ‘end malnutrition’ by 2030 [7].

All South Asian countries are committed to achieving the Global Nutrition Targets by 2025 [8] which are aligned with achieving the SDG 2.2 of, “ending all forms of malnutrition,” by 2030. The six concrete quantified global nutrition targets to accelerate improvement in all nutrition indicators include: (i) 40% global reduction in the number of stunted children; (ii) 50% reduction in anaemia in women in productive age; (iii) 30% reduction in low birth weight; (iv) no increase in childhood overweight; (v) increase the rate of exclusive breast-feeding in infants’ first six months to at least 50%; and (vi) reduce and maintain childhood wasting to less than 5% [9]. Table 1 provides the prevalence of these nutrition and related indicators across South Asia.

**Table 1:** Nutrition and related indicators in South Asia

	**AFN**	**BNG**	**BHT**	**IND**	**MAL**	**NPL**	**PKT**	**SRL**
Stunting prevalence, children under 5 years of age (%)	35.1 (2020)	30.2 (2020)	22.4 (2020)	30.9 (2020)	14.2 (2020)	30.4 (2020)	36.7 (2020)	16.0 (2020)
Prevalence of anaemia in women of reproductive age (aged 15–49) (%)	42.6 (2019)	36.7 (2019)	38.6 (2019)	53.0 (2019)	52.2 (2019)	35.7 (2019)	41.3 (2019)	34.6 (2019)
Overweight prevalence among children under 5 years of age (%)	3.9 (2020)	2.1 (2020)	5.2 (2020)	1.9 (2020)	4.6 (2020)	1.8 (2020)	3.4 (2020)	1.3 (2020)
Infants exclusively breastfed for the first 6 months of life (%)	57.5 (2018)	62.6 (2019)	53.2 (2015)	58.0 (2017)	63.0 (2017)	62.1 (2019)	47.8 (2018)	80.9 (2016)
Prevalence of wasted children under 5 years of age (%)	5.1 (2018)	9.8 (2019)	5.9 (2010)	17.3 (2017)	9.1 (2017)	12.0 (2019)	7.1 (2018)	15.1 (2016)

Source: WHO Global Health Observatory

*AFN* *Afghanistan, BNG* *Bangladesh, BHT* *Bhutan, IND* *India, MAL* *Maldives, NPL* *Nepal, PKT* *Pakistan, SRL* *Sri Lanka*

**Fig. 1:**
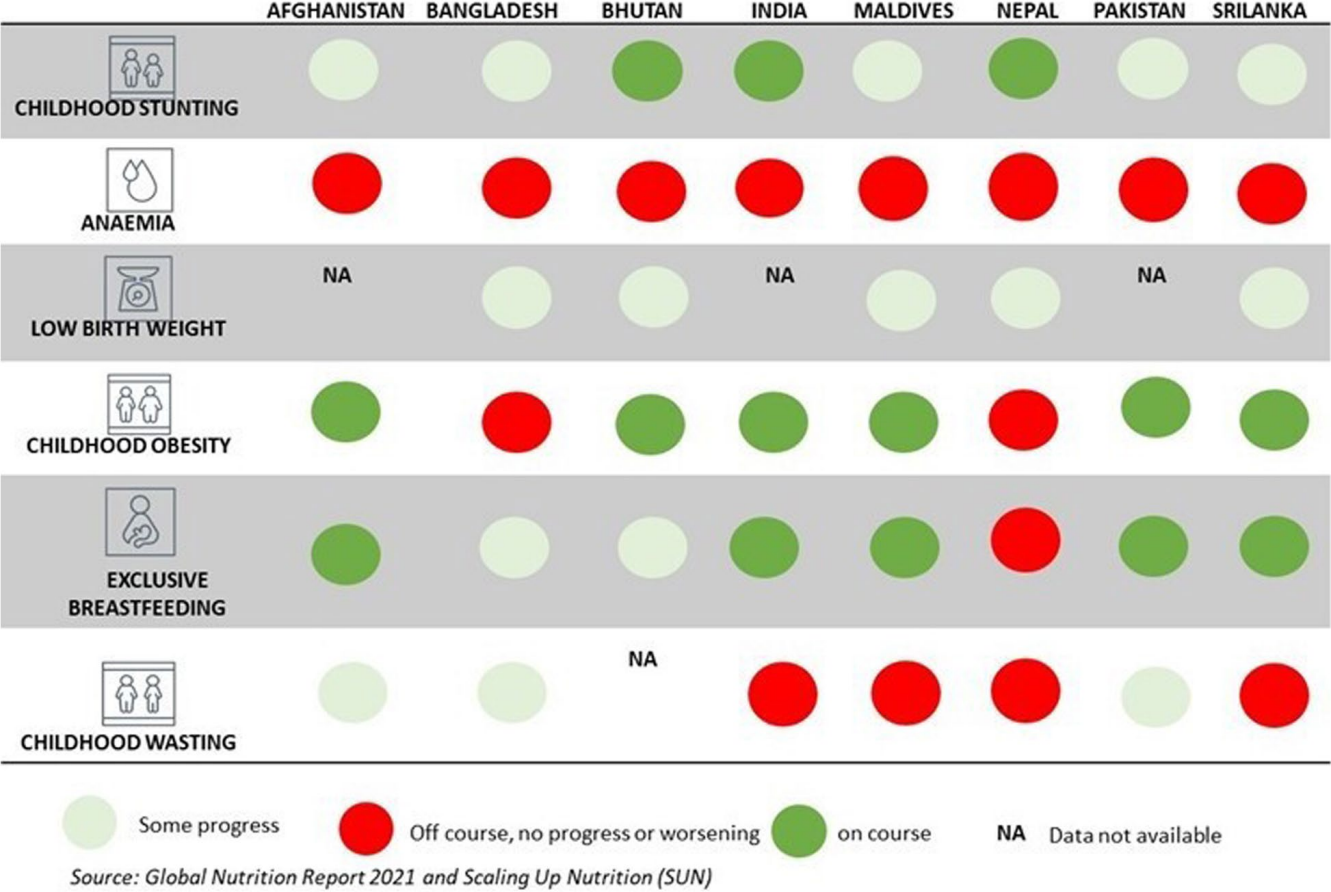
Nutrition progress in South Asia

Based on the latest Global Nutrition Report, most South Asian countries are on track to meet target 1 to reduce child stunting, but there are gaps to achieving all other targets, especially to address anaemia in women which requires tremendous efforts (see Fig. 1). The WHO outlined priority actions and recommended activities to achieve these six Global Nutrition targets. These actions focus on creating a supportive environment for nutrition-sensitive and nutrition-specific investments at policy and program levels through an intersectoral approach. As global recommendations and subsequent priority actions are based on existing evidence, it is anticipated that national governments and relevant stakeholders utilise these recommendations to update national policies and programs. At present there is limited evidence of the policy coherence of South Asia region with globally recommended actions along with how these countries are progressing on their nutrition actions. To fill this gap in literature this study mapped the existing national nutrition policies and programs designed to address child malnutrition, in all its forms, across the eight South Asia countries.

## Methods

We undertook a systematic internet search to map the available national policies and programs in nutrition-specific and nutrition-sensitive sectors to address child malnutrition in eight South Asian countries. Nutrition-specific policies included all governmental policies, programs, legislation, regulations, strategies and plans directly related to nutrition, food security, or food and nutrition security. Nutrition-sensitive policies included those that were indirectly related to nutrition outcomes in children such as agriculture, maternal health, education and nutrition-sensitive interventions in development, poverty reduction, and social protection [10].

The eight South Asian countries included were based on the United Nations regions: Afghanistan, Bangladesh, Bhutan, India, Maldives, Nepal, Pakistan and Sri Lanka [11]. This study looked at all forms of malnutrition including undernutrition- stunting, wasting and underweight, hidden hunger caused by micronutrient deficiencies and overnutrition, each with global significance in South Asia [12, 13].

The term ‘Policy’ is defined as a ‘specific official decision or set of decisions designed to carry out a course of action endorsed by a political body, including a set of goals, priorities and main directions for attaining these goals, including legislation and product reformulation mandates’ [14]. In terms of government actions (which can include national and regional), as defined by the United Nations, ‘policies’ in this context encompasses laws, regulations, social policies, strategies and service provision/ schemes [15].

### Data collection

Data were collected from websites of government departments of health, agriculture, education and labour in the eight target countries. In addition, the websites of key international organisations operating in the countries were searched. These included:

1. Asia Development Bank (ADB),
2. Australia's Department of Foreign Affairs and Trade (DFAT),
3. European Union (EU),
4. Department of Foreign Affairs, Trade and Development Canada (DFATD Canada),
5. Department of International Development, UK (DFID),
6. Food and Agriculture Organisations (FAO),
7. United Nations Development Programme (UNDP),
8. United Nations Children’s Fund (UNICEF),
9. United Nations Office of Coordination of Humanitarian Affairs (UNOCHA),
10. United States Agency for International Development (USAID),
11. World Food Programme (WFP) and
12. World Health Organisation (WHO)

All searches were conducted in English and latest policies related to nutrition-specific and nutrition-sensitive policies were considered for the review. Only official national policy and program documents by the national government and those relevant to national nutrition and related policies/programs, available at the websites of above stated international organisations were included in this study.

### Data mapping and analysis

The authors developed a template to map existing child malnutrition national policies and programs, as presented in Table 2. This template was adapted to align within the nutrition-specific and nutrition sensitive-interventions [16], as specified by the WHO and the United Nations and the priority action list of recommended activities by the WHO [8]. The template was developed by the primary author in consultation with all the authors. All included documents were mapped against the following criteria:

1. Towards Enabling supportive environment for implementation of comprehensive food and nutrition policies: this included all national efforts to formulate national nutrition and food policies along with efforts for intersectoral coordination and stakeholder engagement.
2. Initiatives targeted at Nutrition-Specific interventions: includes all national initiatives that address the immediate determinants of fetal and child nutrition and development.
3. Initiatives targeted at Nutrition-Sensitive interventions: includes all national initiatives outside the health sector that recognize and include nutrition.

**Table 2:** List of documents reviewed for the study

Source (Year)	Type of document	Country	Focus (Outcome)	Mode of Implementation	Funding Pvt, Public or both	M&E framework
1. World Bank, 2021 [18]	Report	Bhutan, India, Nepal, and Sri Lanka	Funding	Not applicable (NA)	Both	NA
2. Govt. of Afghanistan, 2015, [19]	Policy Document	Afghanistan	Health	Co-implementation by govt. along with partner agencies such as FAO, UNICEF, WFP	Both	No
3. Govt. of Afghanistan, 2015, [20]	Strategy document	Afghanistan	Nutrition	Co-implementation by govt. along with partner agencies such as FAO, UNICEF, WFP	Both	Yes
4. Sharma J, Ludin H, Chauhan M, Zodpey S., 2021 [21]	Policy Review	Afghanistan	Nutrition	Co-implementation by govt. along with partner agencies such as FAO, UNICEF, WFP	Both	Yes
5. Scaling Up Nutrition (SUN), 2020 [22]	Country Report	Afghanistan	Nutrition	NA	NA	NA
6. Royal Govt. of Bhutan, 2021 [23]	Strategy document	Bhutan	Food & Nutrition Security	Co-implementation by govt. along with partner agencies such as WFP	Both	Yes
7. Royal Govt. of Bhutan, 2022 [24]	Policy- note	Bhutan	MCH	Co-implementation by govt. along with partner agencies such as WFP	Both	Yes
8. IBFAN, 2017 [25]	Report	Bhutan	IYCF	NA	NA	NA
9. Govt. of the People’s Republic of Bangladesh, 2017 [26]	Policy Document (2016–2025)	Bangladesh	Nutrition	Co-implementation by govt. along with partner agencies such as UNICEF, WHO, WFP, DFAT, US AID	Both	Yes
10. SUN, 2022 [27]	Country Report	Bangladesh	Nutrition	NA	NA	NA
11. Govt. of the People’s Republic of Bangladesh, 2017 [28]	Policy document	Bangladesh	Social Security	Co-implementation by Govt. along with partner agencies such as UNICEF, WHO, WFP, DFAT, US AID	Both	Yes
12. Government of India, 2022 [29]	Policy Document	India	Nutrition	De-centralised through local govts	Both	Yes
13. Lim S, et al., 2009 [30]	Journal Article	India	Cash Transfers	NA	NA	NA
14. Karan A, Negandhi H, Hussain S, Zapata T, 2021 [31]	Journal Article	India	Human Resources	NA	NA	NA
15. Rathi K, Kamboj P, Bansal P, Toteja G. 2018, [32]	Journal Article	India	Health and Nutrition survey	NA	NA	NA
16. Govt. of Maldives, 2016 [33]	Strategy document	Maldives	Nutrition	Co-implementation by Govt. along with partner agencies such as UNICEF	INA	Yes
17. IBFAN, 2016 [34]	Report	Nepal	IYCF	NA	NA	NA
18. National Planning Commission, Nepal 2017 [35]	Policy Document	Nepal	Nutrition in all its forms	Co-implementation by Govt. along with partner agencies such as UNICEF	Both	Yes
19. UNICEF & World Bank, 2013 [36]	Programs Report	Nepal	Nutrition	NA	NA	NA
20. SUN, 2022 [37]	Country Profile	Nepal	Nutrition	NA	NA	NA
21. World Food Programme, 2022 [38]	Web page	Sri Lanka	Food security	Co-implementation by Govt. along with partner agencies such as WFP	INA	INA
22. Asian Development Bank, 2022 [39]	Web page	Sri Lanka	Food security	INA	INA	INA
23. Govt. of Sri Lanka, 2022 [40]	Policy Document	Sri Lanka	Nutrition	Co-implementation by Govt. along with partner agencies such as WFP	Both	Yes
24. SUN, 2021 [41]	Country Report	Pakistan	Nutrition	NA	NA	NA
25. Govt. of Pakistan, 2017 [42]	Policy Document	Pakistan	Nutrition	Co-implementation by Govt. along with partner agencies such as WFP	Both	Yes
26. Cheema I, et al., 2020. [43]	Evaluation Report	Pakistan	Social security	NA	NA	NA
27. World Bank, 2019 [44]	Report	India, Nepal and Bangladesh	BHFI	NA	NA	NA
28. ILO, 2014 [45]	Law and Practices	South Asia	Maternity benefits	NA	NA	NA
29. Codling K, Rudert C, Bégin F, Peña-Rosas JP., 2017 [46]	Journal Article	South Asia	Salt iodisation Legislative Framework	NA	NA	NA
30. Food Fortification Initiative, 2021 [47]	Web page	South Asia	Food Fortification	NA	NA	NA
31. Global Nutrition Report, 2022 [48]	Web page	South Asia	Nutrition	NA	NA	NA
32. UNICEF, 2021 [15]	Policy Research Report	South Asia	Child protection and Family friendly policies	NA	NA	NA
33. UNICEF, 2020 [49]	Research Report	South Asia	Social Protection Programmes	NA	NA	NA

*NA* *Not applicable*

*INA* *Information not available*

*BFHI* *Baby-friendly Hospital Initiative*

A thematic analysis of all the documents that met the operational definitions of a policy or program was undertaken. The analysis involved reading the retained documents to become familiar with their content, coding documents and mapping them against the priority actions and interventions, as outlined in Table 2. The primary author conducted coding and any doubts were discussed with the authors. This method was modelled on Thomas and Harden’s framework [17], to include mapping against priority actions. Information extracted from these documents was based on a snowballing approach where relevant data was extracted from various documents and then reviewed against any additional evaluation report or academic study that might be available for the country. A narrative descriptive approach was used to present the findings.

## Results

The eight South Asian countries included in this review had at least one primary national nutrition policy/program in place. Most countries had a government department or ministry responsible for implementing the nutrition policies and national programs. However, countries such as Afghanistan and Sri Lanka continue to witness war, civil unrest, and political instability which impacts the collection of reliable data for policy analysis. Table 2 provides the list of documents reviewed for the study and Table 3 provides a detailed mapping of national policies based on the activities and recommendations of WHO towards achieving the Global Nutrition Targets. This section begins with a brief introduction to the funding landscape of South Asia, providing a context of the public vs private nutrition funding across these countries, it then describes the nutrition-specific and nutrition-sensitive national policies/programs across South Asia.

**Table 3:** Countries with nutrition-specific and nutrition-sensitive policies corresponding to priority actions in South Asia

	***Priority action***[8]		***AFN***	***BNG***	***BHT***	***IND***	***MAL***	***NPL***	***PKT***	***SRL***
*1*	Creating an Enabling environment for implementation of comprehensive food and nutrition policies	Specific nutrition policies to address undernutrition including stunting, wasting and underweight	✓	✓	✓	✓	✓	✓	✓	✓
		Specific nutrition policies to address micronutrient deficiencies	✓	✓	✓	✓	✓	✓	✓	✓
		Specific nutrition policies to address overweight	╳	✓	╳	✓	✓	✓	╳	✓
		Food and nutrition security policies	╳	✓	╳	✓	╳	✓	✓	✓
		Include nutrition in the development of country’s, poverty reduction and social protection policies	✓	✓	✓	✓	✓	✓	✓	✓
		Established intersectoral coordination mechanism to support food and nutrition security policies	✓	✓	✓	✓	✓	✓	╳	╳
*2*	Initiatives targeted at Nutrition-Specific interventions	Breast-feeding promotion, protection, and support: early initiation, exclusive, counselling and support	✓	✓	✓	✓	✓	✓	✓	✓
		Complementary and Supplementary Feeding	✓	✓	✓	✓	✓	✓	✓	✓
		National guidelines for distribution of micronutrient supplements for children aged 6–23 months	✓	✓	INA	✓	✓	✓	INA	✓
		Interventions for adolescent girls such as: Iron supplementation, WASH practices, management of overweight	✓	✓	✓	✓	✓	✓	✓	✓
		Interventions for pregnant women	✓	✓	✓	✓	✓	✓	✓	✓
		Distribution of micronutrients powder and micronutrient supplementation norms	✓	✓	✓	✓	✓	✓	✓	✓
*3*	Initiatives targets at nutrition-sensitive interventions	Policies on smallholder agriculture	╳	✓	✓	✓	INA	INA	INA	INA
		School-based nutrition programs	✓	✓	INA	✓	INA	✓	INA	INA
		Policies on appropriate nutrition in food manufacture, food labelling norms	✓	✓	INA	INA	INA	INA	INA	INA
		Policies to promote healthy diets and healthy lifestyles	✓	✓	INA	INA	INA	INA	INA	INA
		Policies on WASH with a focus on nutrition	╳	✓	╳	✓	✓	✓	✓	✓
		Improved work environment to support women with children	╳	INA	INA	✓	INA	INA	INA	INA
		Regulation of marketing of sugar-sweetened beverages and energy-dense nutrient-poor products and fast foods	✓	✓	INA	✓	INA	INA	INA	INA
		Policies on maternal/women's education	╳	✓	✓	✓	✓	✓	✓	✓
*4*	Monitoring & Evaluation	Existence of implementation framework	✓	✓	✓	✓	✓	✓	✓	✓
		Monitoring and Evaluation framework	✓	✓	✓	✓	✓	✓	✓	✓

*Source:* Prepared by the authors based on the study results

Nutrition-related policies were defined as “all governmental policies, legislation, regulations, strategies, plans, clinical norms, or clinical guideline related directly or indirectly to nutrition, food security, or food and nutrition security”; sectoral policies included those related to agriculture, food, maternal and child nutrition, health, education, and physical activity and nutrition-sensitive interventions in development, poverty reduction, and social protection

World Health Organization Comprehensive Implementation Plan on Maternal, Infant and Young Child Nutrition [8]

*AFN *Afghanistan, *BNG *Bangladesh, *BHT* Bhutan, *IND* India, *MAL* Maldives, *NPL* Nepal, *PKT* Pakistan, *SRL* Sri Lanka

✓: Yes; ╳: No policy; *INA* Information not available

### Nutrition funding landscape in South Asia

Public expenditures varied widely between countries based on spending per capita, as a percentage of gross domestic product (GDP), and as a percentage of general government expenditure (GGE). Expenditure on nutrition-sensitive programs was higher than nutrition-specific initiatives; this could be as the entire expenditures of nutrition-sensitive interventions were accounted as nutrition-related while these interventions typically have an indirect impact on nutrition. For instance, smallholder agriculture or Water Sanitation and Hygiene (WASH) intervention will impact on multiple sectors along with nutrition. They were wide discrepancies across countries on nutrition-sensitive spending across Bhutan (2017), Nepal (2016), and Sri Lanka (2015). Spending on nutrition-specific interventions was between 4 and 28 percent of overall nutrition spending in Bhutan, Nepal, and Sri Lanka [18].

**Fig. 2:**
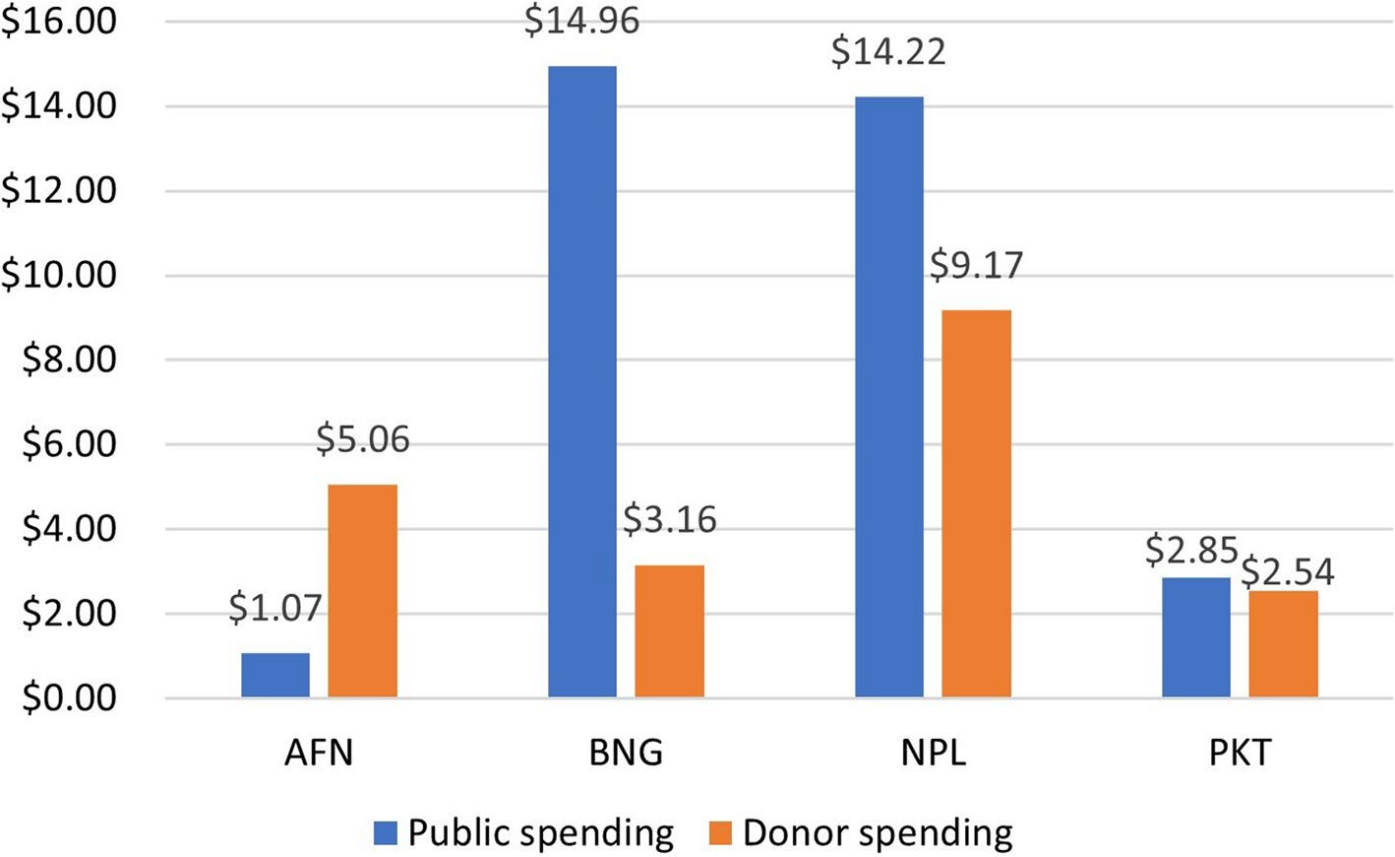
Per child spending on Nutrition-specific interventions, for children U5

Data on nutrition investment and spending were patchy across countries which made it difficult to understand budget allocation and expenditure specific to nutrition. This was further exacerbated as nutrition-related activities across South Asian countries were bundled with health service packages across multiple ministries [18]. There is also limited data on funds utilised for nutrition and evaluating implementation across interventions. Based on the data from SUN country reports, spending on nutrition-specific interventions varied across four South Asian countries. Bangladesh and Nepal public spending was more than donor spending, while Afghanistan had more donor spending than public spending, as illustrated in Fig. 2. Data for other countries were unavailable.

### Action 1. Creation of an enabling environment for the implementation of comprehensive food and nutrition policies

The Right to Food is recognised in the constitution of Bangladesh, India, Maldives, Nepal, Pakistan and Sri Lanka. Afghanistan and Bhutan did not explicitly recognise the Right to Food but had provisions in place for food security.

All South Asian countries had comprehensive food and nutrition policies to address child undernutrition. These policies included programs and interventions that address specific forms of undernutrition, improve infant and young child feeding practices and prevent micronutrient deficiencies. Afghanistan had the National Public Nutrition Policy and Strategy (2015–2020) in place and was committed to reducing stunting in children under 5 years of age to 10% by 2030 [19]. India’s Integrated Child Development Services or ICDS Scheme was the world’s largest and unique early childhood care and development programs. The ICDS aimed to improve the nutrition status and overall development of children under the age of 6 years and the nutrition of pregnant and lactating women (PLW) [29]. Bangladesh’s Second Action Plan for Nutrition committed to delivering on nutrition targets of global commitments to SUN and WHA [26]. The Government of Nepal implemented the Multi-Sector Nutrition Plan MSNP II (2018–2022) targeted to reduce intergenerational transmission of stunting [36]. Additionally, Nepal had undertaken a variety of measures to address the country’s nutritional concerns and presently implemented the National Nutrition Program (2018–22). Pakistan is committed to achieving global nutrition targets under its Multi-sectoral Nutrition Strategy (2018–2025) [41]. Plans or strategies to address micronutrient deficiencies included diet diversification, distribution of micronutrient powder, micronutrient supplementation and food fortification. Bangladesh, India and Nepal had robust strategies to address micronutrient deficiencies. Table 4 below provides the country-wise details of distribution of micronutrient powder and supplementation norms across South Asia.

**Table 4:** Distribution of micronutrients powder and micronutrient supplementation norms across South Asian countries

	AFN	BNG	BHT	IND	MAL	NPL	PKT	SRL
Vitamin A suppl., 6–59 months old	✓	✓	✓	✓	✓	✓	✓	✓
Daily IFA, nonpregnant women	✓	✓		✓	✓	✓	✓	
Intermittent IFA suppl. for nonpregnant women				✓		✓	✓	
Daily IFA suppl. for pregnant women	✓	✓	✓	✓	✓		✓	✓
Zinc suppl. for management of diarrhea	✓	✓		✓	✓	✓	✓	✓
Calcium suppl. during pregnancy		✓		✓				✓

All countries except Afghanistan and Bhutan had policies to address obesity and overweight [27, 50]. Bangladesh, India, Maldives and Sri Lanka each had multisectoral action plans to prevent and control non-communicable diseases. These policies aimed to reduce preventable morbidity, avoidable disability and premature mortality with components to promote physical activity, minimise consumption of saturated fatty acids and promote the intake of healthy foods.

South Asian countries established intersectoral coordination mechanisms to support food and nutrition security policies. Afghanistan had AFSeN-A, a Food Security and Nutrition Steering Committee and Executive Committee [22], Bangladesh established the Bangladesh National Nutrition Council [27] and Bhutan’s Food and Nutrition Security policy (2014) was managed by Ministry of Agriculture and Ministry of Health. In Nepal, High-Level Nutrition and Food Security Steering Committee was the highest governing body, providing guidance and endorsing policies and programs. The National Nutrition and Food Security Coordination Committee coordinated and provided operational guidance for Multi-Sector Nutrition Plan (MSNP II) (2018–2022) implementation [37].

#### Food and Nutrition security programs

India’s Public Distribution System was a fully government funded program, the largest food distribution program in the world with the highest coverage of two third of its population [49]. It distributes food and non-food items including of wheat, sugar, rice and kerosene (can differ between states) to poor people and those in need at subsidised rates. In 2009, Nepal introduced the Child Grant under which mothers of eligible children, under five years of age, received a quarterly unconditional cash transfer of US$14 to cover their basic needs [15]. This program originally targeted populations living in remote and mountainous region but was recently expanded to becoming universal, with support from UNICEF. Pakistan’s Benazir Income Support Program or BISP, primarily funded by the Government of Pakistan, was the largest cash transfer program in South Asia. It provided unconditional cash transfers to extremely poor families- where the beneficiaries were exclusively women [43]. Lastly, Sri Lanka’s Samurdhi program provided cash transfers to poor families, it was the largest safety net program to protect families in emergencies [38]. The Samurdhi program was fully funded by the Sri Lankan government until the recent civil unrest, which has seen investment from International donors such as the Asian Development Bank [39].

### Action 2. Policies and programs targeted at nutrition-specific interventions

All South Asian countries have breastfeeding laws, where they adopted provisions of WHO’s International Code of Marketing of Breast-milk Substitutes, except Bhutan which has voluntary guidelines [25]. Most countries policies protect, promote and support optimal infant young child feeding practices (IYCF) (Afghanistan, Bangladesh, Maldives, India, Pakistan and Sri Lanka) except Bhutan which has no Information Education and Communication (IEC) strategy to improve IYCF [25] and Nepal’s national IEC materials were inadequate with poor geographical coverage [34].

In all countries except for Bangladesh and Pakistan, mothers are guaranteed breastfeeding breaks by law, although the period of entitlement varies across countries [15]. Four of the eight South Asian countries (Afghanistan, Bangladesh, India and Pakistan) mandated employers under labour policies and legislative frameworks to ensure on-site childcare provision at the workplace. However, Bangladesh, India and Pakistan require a minimum threshold of women employees to trigger this enforcement [15]. All countries had adopted the Baby-friendly Hospital Initiative (BFHI), while India, Maldives and Nepal struggled with the implementation and Bhutan had not yet implemented the initiative [44].

South Asian countries of Bangladesh, Bhutan, India, Nepal and Sri Lanka had schemes that provided cash transfers, and maternity benefits to PLW. Bangladesh’s Maternity Allowance for the Poor Lactating Mothers provided monthly cash transfers to poor mothers during their lactating period for up to three years. Eligibility to receive the benefit were women’s age (20 years and above) and only for the first two children [28]. Janani Suraksha Yojana or JSY in India provided cash transfers to poor PLW, conditional upon either in-facility delivery or the attendance of skilled healthcare personnel at birth [30]. To complement the JSY, India recently implemented a cash transfer for mothers during their breastfeeding period, the Pradhan Mantri Matru Vandana Yojana or PMMVY. PMMVY targeted all first-time pregnant women over 18 years of age and lactating mothers (except those working in the public sector or receiving similar benefits). Nepal’s Aama or Safe Motherhood Program was a universal program that allowed beneficiaries deliver for free in a health facility (for their first two children) and provided cash transfers to pregnant women to pay for transportation to attend four prenatal visits and one postnatal visit. In Sri Lanka, the National Supplementary Food Program or Thriposha provided supplementary foods to all PLW, as well as children under 5 years of age identified as malnourished [38]. This scheme gave all beneficiaries 10 vouchers to be spent on food, distributed over 10 months from the third month of pregnancy until the child is 4 months old [38]. Bhutan recently started the Accelerating Mother and Child Health Program or AMCH that provided cash incentives for mothers who avail the requisite maternity and child health services [24].

All eight countries adopted national legislation for paid leave to mothers of newborns. However, only India and Bangladesh complied with the International Labour Organisation (ILO) standard of 14 weeks of maternity leave. India and Nepal had maternity leave programs financed through social security contributions (through the employee and employer) which were government- administered while other countries had the ‘employer liability system’ that made the employers liable for paying the maternity benefit [15]. Only Bhutan, Maldives and Nepal adopted paternity leave legislation, that allowed paternity leave for five, three and 15 days [45]. Notably, the maternity and paternity leave benefits were applicable to the formal employment sector and excluded women working in the informal and agriculture sectors, leading to low coverage of these benefits.

All South Asian countries had a legislation for mandatory salt iodisation except for Bhutan and Maldives [14]. However, the Bhutanese government banned the import of non-iodised salt since 1984 and imported only iodised salt from India [46]. Maldives imported most of its salt with a planned focus on strengthening the quality assurance of imported salt [46]. Other food fortifications included: wheat flour fortification with iron, zinc, vitamin A, vitamin B6 and B12, folic acid, niacin, thiamin, and riboflavin. Afghanistan and Nepal had mandatory legislation for wheat flour fortification while in Bangladesh and India it was voluntary. Other South Asian countries of Bhutan, Maldives, Sri Lanka and Pakistan had no wheat flour fortification legislation [47]. Rice fortification with iron, zinc, vitamin B6, B12, folic acid, niacin, thiamine, riboflavin, Vitamin A. No South Asian country had mandatory legislation for rice fortification except for Bangladesh and India where it was voluntary [47].

### Action 3. Policies and programs targeted at nutrition-sensitive interventions

All countries except Bhutan, Maldives and Pakistan had food-based dietary guidelines (FBDGs) that complied with the joint Food and Agriculture Organization (FAO)/WHO consultation on the preparation and use of FBDGs. Bangladesh, India and Sri Lanka had sugar-sweetened beverage tax. India and Sri Lanka had policies to reduce salt/sodium consumption. Only India had the policy to limit saturated fatty acid intake and to eliminate industrially produced trans fatty acids. Nepal, India and Sri Lanka adopted policies to reduce the impact of marketing of foods and beverages high in saturated fats, trans fatty acids, free sugars, or salt on children. All South Asian countries except Pakistan had an operational policy, strategy, or action plan to reduce unhealthy diets related to non-communicable diseases and diabetes [48].

All countries had school-based nutrition programs including feeding programs to address nutrition and encourage school attendance. The WFP implemented feeding programs in Afghanistan and in conflict and disaster-affected areas in Pakistan. Information on a universal school-based feeding program in Pakistan was unavailable.

All countries had policies to promote sustainable agriculture practices such as family farming and smallholder farming, targeted to address food diversity and food security and build resilience to disasters and climate change. All countries had policies to promote women’s education and to discourage school dropouts except Afghanistan, where information was unavailable. All countries had water and sanitation policies, but these were not necessarily linked to nutrition outcomes. However, nutrition-sensitive interventions were designed to include WASH elements in it.

#### Human resources for nutrition interventions

Data on nutrition professionals was not available, health professionals including community health workers were assumed to take on the work required by nutrition professionals. The density of physicians per 10,000 people was 2.6 for Afghanistan, 3.6 for Bangladesh, 2.6 for Bhutan, 8.8 for India [31], 45.6 for Maldives, 7.5 for Nepal, 9.8 for Pakistan and 1.0 for Sri Lanka. The density of nurses and midwives were 2.2 for Bangladesh, 5.5 for Afghanistan, 9.9 for Bhutan, 17.7 for India [31], 64.3 for Maldives, 31.1 for Nepal, 6.7 for Pakistan and 21.8 for Sri Lanka. Density of Community health workers was 0.7 for Afghanistan, 3.3 for Bangladesh, 8.5 for Bhutan, 14.2 for Maldives, 6.8 for Nepal and 0.9 for Pakistan [48].

### Action 4: Monitoring and evaluation mechanisms of the implementation of policies and programs

All South Asian countries adopted the WHO growth standards. Countries in the region collected information through international surveys such as Demographic Health Surveys and have recently seen data collected by the government with UNICEF for the National Nutrition Survey and National Micronutrient Status Survey. Additionally, Bangladesh collected nutrition data from several sources, such as the Nutrition Information System and Nutritional Information Planning Unit, District Health Information Software, the Food Security and Nutrition Information System of FPMU, Food Security Nutritional Surveillance Program [26]. India conducted various surveys National Family Health Surveys, District Level Household Survey, Annual Health Survey, National Nutrition Monitoring Bureau Survey, Rapid Survey on Children and Comprehensive National Nutrition Survey [32]. Afghanistan under the National Public Nutrition Policy and Strategy (2015–2020) was to establish the Nutrition Monitoring and Surveillance System to track and assess the quality, coverage and impact of public nutrition interventions. However, with the political instability there was no information available on its implementation. All South Asian countries had an implementation framework and committees responsible for the nutrition strategy but there was limited information in the policy documents of the actual implementation and its gaps. All countries mentioned having a Monitoring and Evaluation framework but there was limited information on its specific components.

## Discussion

South Asian (SA) countries have developed and established comprehensive national policies and programs to address all forms of child malnutrition. However, despite the efforts micronutrient deficiencies, anaemia, wasting and being overweight continue to pose public health concern in the region [51]. This study mapped the nutrition-specific and nutrition-sensitive sectoral policies and programs of eight SA countries.

Findings suggest that SA countries established an enabling environment to support food and nutrition policies and programs along with other related sectoral policies/programs to address child malnutrition. For instance, the right to food is recognised in the Constitution for all SA countries except Afghanistan and Bhutan. All countries had nutrition actions incorporated in policies or programs of related sectors of health, agriculture, education, and social protection. Conditional cash transfers for first-time mothers and to ensure food security within households was implemented in most SA countries but there continue to be issues of outreach and utilisation. Evaluations of such schemes reveal low benefit adequacy with low impacts and high exclusion errors due to poor targeting [15].

Nutrition-sensitive interventions that address underlying determinants of undernutrition to improve household wealth, improving access to water, sanitation, health services, purchasing capacity, and women’s education were incorporated across all countries. However, there was limited information on the effectiveness of these interventions on nutrition due to limited program and policy evaluation data [16]. Research suggests nutrition-sensitive programs can have an enhanced impact by improved targeting, strengthening of nutrition goals, design, and implementation along with a focus on women’s nutrition, time, physical and mental health, and empowerment [16].

South Asia made significant efforts to prevent micronutrient deficiencies and developed national plans, including food fortification guidelines for distribution of micronutrient powder and micronutrient supplementation. However, the evidence was patchy around their implementation and effectiveness. In Nepal the female community health workers volunteers program successfully reached out to provide behaviour change counselling and Vitamin A and Fe supplements [52]. Nepal’s Vitamin A program achieved 88% coverage and was recognised as a success by the global community [53]. India’s Vitamin A supplementation program had poor coverage for children from remote and vulnerable communities of scheduled castes and scheduled tribes [53]. Improved coverage targeting such groups is vital for population-level impact to address micronutrient deficiencies in a population. Most SA countries had legislation of iodisation of salt except for Bhutan which banned the import of non-iodised salt and Maldives with a commitment to improving the quality of iodised salt. Research suggests that successful implementation of these initiatives steadily reduced the risk of Iodine deficiency disorders in the region [46]. Other food fortifications of wheat and rice were voluntary in most countries which might have contributed to the existing micronutrient deficiencies in the region. However, efforts to enrich and fortify staple South Asian foods with Fe, vitamin A, Zn and other micronutrients were underway with a potential to substantially reduce micronutrient deficiencies in the region [51].

Overweight and obesity is of growing concern across the SA countries. While the proportion of the obese population was not found to be alarming at present, it was increasing steadily and efforts to prevent obesity were not at par to address the issue. While most countries had a policy to reduce unhealthy diets related to non-communicable diseases and diabetes [48], only a few established various regulatory frameworks to address overweight and obesity. For instance, only Bangladesh, India and Sri Lanka implemented food-based dietary guidelines and taxation of sugar-sweetened beverages; India and Sri Lanka had policies to reduce salt/sodium consumption and only India had a limit to saturated fatty acid intake and to eliminate industrially produced trans fatty acids. Nepal, India and Sri Lanka had policies to reduce the impact of marketing of foods and beverages high in saturated fats, trans fatty acids, free sugars, or salt on children.

All countries introduced policies and interventions to improve infant and young child feeding practices and enacted the provisions of WHO’s International Code of Marketing of Breast-milk Substitutes [54], except Bhutan [25]. Interventions to promote breastfeeding, timely and age-appropriate complementary feeding and dietary diversity are necessary to combat micronutrient deficiencies and undernutrition including stunting and wasting. Breastfeeding is also increasingly recognised as a protective agent against overweight and obesity [55].

Most SA countries enforced laws to ensure breastfeeding mothers had breaks during work except Bangladesh and Pakistan, although the period of entitlement varied across countries. All countries adopted national legislation for paid leave to mothers of newborns [15]. While this helps mothers and caregivers to reconcile work and family, in SA it needs to be informed by the pattern of labour. Majority of workforce in SA is employed in informal or agriculture sectors and these policies are often relevant for the formal sector, leading to low coverage of benefits. Informal work is associated with unhealthy conditions, lower and volatile salaries and lacks bargaining power to claim basic rights [49]. There is a need to extend the discourse around family-friendly policies beyond the formal workplace in SA. While Baby-friendly Hospital Initiative (BFHI) was adopted across SA countries, implementation was a challenge in Bhutan, India, Maldives and Nepal [44].

Most SA countries had a shortage of trained human resources in the health and nutrition sector. In all South Asian countries, no data were available for personnel working in the nutrition sector in which case, health workers were assumed as proxy nutrition workers. The density of health workers per 10,000 was much below the WHO threshold of 44.5 nurses/doctors [56] with skewed health workforce distribution within each country. Despite the increased recognition of health workers’ role in achieving health outcomes and enhanced economic growth there continues to be a low investment in education and training of the health workforce in most lower and middle-income countries (LMICs) [57]. While most countries allocated budget for nutrition programs including nutrition-specific and sensitive interventions. Research reveals substantial challenges in tracking and monitoring these expenditures, as multiple ministries were responsible for nutrition-related activities. For instance, interventions to improve dietary diversity may be linked to agricultural production [58]. To address these challenges, many LMICs have been implementing the Scaling Up Nutrition (SUN) movement methodology since 2015, which has allowed to streamline the process [7]. The region also suffers from bureaucracy and corruption which poses a risk of benefits of national schemes not reaching the beneficiaries.

To achieve the Global nutrition targets, there is a need for improved budgetary allocations to nutrition-specific and sensitive interventions [16]. However, for countries such as Nepal and Pakistan there is an increased dependency on international donors and organisations for funding and/or implementation of nutrition plans which puts into question long term sustainability of interventions. While countries such as Afghanistan and Sri Lanka with the ongoing war and civil unrest make the role of international organisations inevitable and crucial for resources to reach to women and children. Further there is a need to contextualise costs and implementation efforts designated to donors and governments to improve the tracking of investments and impacts of nutrition.

This study found a lack of focus on interventions and policies that address factors such as gender and social-culture factors and norms, including patriarchy. More so existing interventions did not focus on specific factors that contribute to low female participation in education, maternal mental health and women’s autonomy and agency. South Asian countries will benefit from implementing nutrition behavioural change interventions at the national level that address gender, culture, and social norms along with the existing nutrition-specific and sensitive interventions [59]. Behavioural change interventions are increasingly recognised to influence behaviours and attitudes and can have long term positive impact on health and nutrition of women and children [60].

This study has strengths and limitations worth highlighting. This is the first study that brings together nutrition-specific and nutritional-sensitive government policies and programs across the eight South Asian countries. It allows mapping the national initiatives by the South Asian governments to achieve the Global Nutrition targets to understand country wise progress and commitments to fight child undernutrition. However, this study did not aim to include evaluations of these policies and programs’ implementation to estimate their outreach and impact. While, the study describes the country wise finances for nutrition it did not map individual funders and stakeholders involved in implementing these policies. Lastly, the study was limited to only national initiatives and policies/programs and interventions of the state or local authorities were not included in the study.

## Conclusions

Most South Asian countries had an agenda to address malnutrition in all its forms, they have developed integrated policies to promote nutrition through multi-sectoral involvement and collaboration. The region has witnessed significant reductions in stunting and improved breastfeeding and significant efforts to address the growing micronutrient deficiencies. Despite these successes child malnutrition continues to pose a public health concern in the region [51]. Overweight and obesity are a growing concern in South Asia, while the policy and program efforts are not at par to combat the situation. National policies and plans lay out a broad range of interventions to prevent and treat malnutrition with a focus on nutrition-specific and sensitive interventions but there are major challenges of implementation, monitoring, evaluation, utilisation, equity, quality of care and sustainability. There is also a need to integrate social factors such as gender and social-culture norms, including patriarchy that act as barriers to child undernutrition, in mainstream nutrition interventions.

### Supplementary Information

**Additional file 1. **Aims and scope statement.

## Data Availability

All data generated or analysed during this study are included in this published article.

## Abbreviations

ADB: Asia Development Bank
BFHI: Baby-friendly Hospital Initiative
CIP: Comprehensive implementation plan
DFAT: Australia's Department of Foreign Affairs and Trade
GDP: Gross Domestic Product
GGE: General Government Expenditure
IEC: Information Education and Communication
IYCF: Infant Young Child Feeding Practices
ILO: International Labour Organisation
LMICs: Lower and Middle-Income countries
PDS: Public Distribution System
PLW: Pregnant and Lactating women
SA: South Asian
SDGs: Sustainable Development Goals
SUN: Scaling Up Nutrition
UNICEF: United Nations Children’s Fund
WASH: Water Sanitation and Hygiene
WFP: World Food Programme
WHA: World Health Assembly
WHO: World Health Organisation
