# Supplementary material for: Mapping of nutrition policies and programs in South Asia towards achieving the Global Nutrition targets
Source: Arch Public Health. 2023 Sep 19;81:171. doi: 10.1186/s13690-023-01186-0 (PMC10507966; doi:10.1186/s13690-023-01186-0)
Supplement: Supplementary file 1 — Additional file 1. Aims and scope statement. [file 13690_2023_1186_MOESM1_ESM.docx]

**Aims and Scope statement**

1. What is known?

South Asia continues to host triple burden of child malnutrition with high levels of child undernutrition, hidden hunger (micronutrient deficiencies) and childhood overweight/obesity. To combat malnutrition the international community along with the National governments have launched initiatives to track the country’s progress towards achieving the Global Nutrition targets by 2025. While existing literature focuses on the progress towards achieving these targets in South Asia, there is little evidence that captures a country’s efforts and initiatives towards achieving the Global Nutrition targets. There is a need to map the existing efforts of nutrition-specific and nutrition-related sectoral programs and policies in South Asia.

1. What does the study add?

This is the first study that brings together nutrition-specific and nutritional-related sectoral government policies and programs across the eight South Asian countries. It allows mapping the national initiatives by the South Asian governments to achieve the Global Nutrition targets by 2025. The authors developed a template to map the existing national policies and programs that directly and indirectly impact child malnutrition. This template is adapted to align within the nutrition-specific and nutrition sensitive-interventions, as specified by the World Health Organization (WHO) and the United Nations and the priority actions list of recommended activities by the WHO. Study findings suggest that while all South Asian countries had an agenda to address malnutrition in all its forms, major challenges of implementation, monitoring, evaluation and quality persisted- with increased dependency on international donors and organisations for funding and/or implementation of nutrition plans. Findings highlight the need to contextualise efforts designated to donors and governments to improve the tracking of efforts that impact nutrition. Additionally, countries need to mainstream context-specific nutrition behavioural change along with nutrition-specific and sensitive interventions aimed at targeting gender and socio-cultural factors that act as barriers to child undernutrition.

1. What are implications for clinical practice, public health and / or research?

This study brings together nutrition program and policy related data for eight South Asian countries. It generates evidence to inform country wise efforts towards the Global Nutrition Targets in 2025 and the Sustainable Development Goals in 2030. It highlights the challenges of program and policy implementation. The study points the need for countries to mainstream context-specific nutrition behavioural change along with nutrition-specific and sensitive interventions and consider the role of gender inequality that acts as barriers to child malnutrition. Finally, it provides a template that can be a useful tool for public health researchers to track a country’s efforts in nutrition policy and practice.
